# A Novel Approach to Anticancer Therapy: Molecular Modules Based on the Barnase:Barstar Pair for Targeted Delivery of HSP70 to Tumor Cells

**Published:** 2018

**Authors:** A. M. Sapozhnikov, A. V. Klinkova, O. A. Shustova, M. V. Grechikhina, M. S. Kilyachus, O. A. Stremovskiy, E. I. Kovalenko, S. M. Deyev

**Affiliations:** Shemyakin – Ovchinnikov Institute of Bioorganic Chemistry RAS, Miklukho-Maklaya Str., 16/10, Moscow, 117997, Russia

**Keywords:** cancer immunotherapy, NK cells, 70 kDa heat shock protein, targeted delivery, HER2/neu antigen, mini-antibody, barnase:barstar

## Abstract

One important distinction between many tumor cell types and normal cells consists in the translocation of a number of intracellular proteins, in particular the 70 kDa heat shock protein (HSP70), to the surface of the plasma membrane. It has been demonstrated that such surface localization of HSP70 on tumor cells is recognized by cytotoxic effectors of the immune system, which increases their cytolytic activity. The mechanisms behind this interaction are not fully clear; however, the phenomenon of surface localization of HSP70 on cancer cells can be used to develop new approaches to antitumor immunotherapy. At the same time, it is known that the presence of HSP70 on a cell’s surface is not a universal feature of cancer cells. Many types of tumor tissues do not express membrane-associated HSP70, which limits the clinical potential of these approaches. In this context, targeted delivery of exogenous HSP70 to the surface of cancer cells with the aim of attracting and activating the cytotoxic effectors of the immune system can be considered a promising means of antitumor immunotherapy. Molecular constructs containing recombinant mini-antibodies specific to tumor-associated antigens (in particular, antibodies specific to HER2/neu-antigen and other markers highly expressed on the surface of a wide range of cancer cells) can be used to target the delivery of HSP70 to tumor tissues. In order to assess the feasibility and effectiveness of this approach, recombinant constructs containing a mini-antibody specific to the HER2/ neu-antigen in the first module and HSP70 molecule or a fragment of this protein in the second module were developed in this study. Strong selective interaction between the modules was ensured by a cohesive unit formed by the barnase:barstar pair, a heterodimer characterized by an unusually high constant of association. During testing of the developed constructs in *in vitro *models the constructs exhibited targeted binding to tumor cells expressing the HER2/neu antigen and the agents had a significant stimulating effect on the cytotoxic activity of NK cells against the respective cancer cells.

## INTRODUCTION

The search for novel approaches to cancer immunotherapy remains relevant, although a large number of studies have focused on this problem [1-3]. One of the reasons why malignant neoplasms emerge and develop in the organism is that the surface of tumor cells is devoid of antigens that can activate the cytotoxic effectors of the immune surveillance system which are responsible for the elimination of transformed cells. In this context, targeted modification of a tumor cell’s surface with molecular structures that are recognized by natural killer cells and, thus, induce a cytolytic response is one of the promising approaches to antitumor immunotherapy. It has recently been demonstrated that heat shock proteins (HSPs) with a molecular weight of 70 kDa (HSP70) are among such structures.

The family of heat shock proteins includes a wide range of highly conserved intracellular proteins which are characterized by both heterogeneous physicochemical properties and a variety of functions. HSPs are expressed in all cell types; various damaging agents can increase their expression level manifold. An elevated intracellular level of HSPs is the universal protective response of cells, which is associated with the unique ability of these proteins to prevent stress-induced aggregation of intracellular proteins and their denaturation, as well as to ensure repair of partially damaged proteins or their proper elimination if irreversible damage occurs. The listed functions and involvement in the folding of newly synthesized polypeptides and transport of intracellular proteins are referred to as the so-called “chaperon” properties of the constitutive pool of HSPs, which is expressed in cells under normal physiological conditions in the absence of stress [4, 5]. However, localization of HSPs is not confined to the intracellular space. In a large series of studies, these proteins were found on the cell surface. In particular, surface HSPs were detected on the plasma membrane in normal [6, 7] and tumor cells [8-14], virus-infected lymphocytes [15], and apoptotic T cells [16-18]. It was demonstrated that HSPs with various molecular weights are expressed on the cell surface, but that surface localization is most typical of 70 kDa HSPs (HSP70). The phenomenon of unusual surface expression of HSPs was described not only for *in vitro *cultured cells, but also for the cells of different patient-derived tissues [12, 14].

The functions of HSPs exposed on the cell surface remain virtually unstudied. At the same time, a hypothesis has been suggested that these cell surface proteins are immunologically important, as their emergence on the plasma membrane can be a signal for the immune system to activate cytotoxic effectors and ensure the elimination of infected, transformed, and damaged cells [19]. Indeed, it is well known today that different subpopulations of T cells and NK cells are capable of recognizing highly conserved determinants of various HSPs. In particular, recognition of the membrane-resident HSP70 and Grp75 by γδ-T cells is MHC-nonrestricted [20]; recognition of Hsp70 (the inducible form of HSP70) by NK cells is also MHC-nonrestricted [21, 22]. Surface-resident HSPs of tumor cells attract NK cells: their count can increase up to 500-fold in tumors expressing these proteins on their surface [23]. Data in the literature is indicative of *in vitro *MHC class I-restricted recognition of Hsp70 by human NK cells on the surface of human K562 erythroleukemia cells and human sarcoma cells exposed to heat shock [24]. It was also demonstrated that surface HSP70 proteins cause a strong humoral and cell-mediated adaptive immunity response. According to a number of studies, HSP70 can be attributed to tumor-associated antigens recognized by various types of T cells, such as CD4- CD8- [25], αβ- and γδ-lymphocytes [26, 27], and natural killer (NK) cells [10, 11, 21]. The recognition of both the constitutive and inducible forms of HSP70 by MHC-restricted and nonrestricted immune cells indicates that surface HSP70 proteins play a crucial role in antitumor immune responses. Based on this fact, a model of immune surveillance was suggested where these cells ensure the first line of defense against infectious agents carrying HSPs on their surface, protect against virus-infected or transformed cells, and against damaged autologous cells. The lymphocyte pool recognizing conserved HSPs is probably induced during ontogenesis as the skin and intestinal microflora develop. The periodic reactivation of these lymphocytes can be caused by common viral and bacterial infections, as well as various stressful stimuli [19].

Application of HSP70 in antitumor therapy attracted the attention of researchers exploring various approaches to this problem [28-32]. However, most of these approaches are based on the ability of HSP70 to form strong complexes with tumor-specific peptides, rather than on direct recognition of membrane-associated HSP70 by cytotoxic effectors of the immune system. This is possibly related to the fact that *in vivo *expression of these proteins on cancer cells is observed not in all types of tumor tissues. This circumstance serves as the basis for the assumption that induction of HSP70 translocation onto the surface of tumor cells or targeted delivery of these molecules into malignant neoplasms to attract and activate cytotoxic immune effectors of the immune system is a new, promising direction in antitumor immunotherapy [33].

It has been recently found by a number of researchers and in our preliminary studies that both full-length HSP70 molecules and synthetic analogues of some HSP70 fragments exhibit an activating effect on natural killer cells. In particular, addition of synthetic HSP70 fragments to a human NK cell culture significantly stimulated the production of IFN-γ by natural killer cells, identically to how this took place in the experiments with recombinant HSP70 [34, 35]. Therefore, HSP70 molecules and fragments of this protein can be regarded as promising structures to be used in bioengineering approaches to the fabrication of molecular constructs for targeted modification of the surface of tumor cells in order to potentiate the antitumor cytotoxic immune response. Targeted delivery of such “cytolytic markers” can be performed by incorporating recombinant mini-antibodies against tumor-specific antigens into the recombinant construct module being designed. In particular, antibodies specific to the HER2/neu antigen (p185HER2) or to other cancer markers expressed on the surface of a wide range of malignant neoplasms can be used as such mini-antibodies.

This study was aimed at developing a method for targeted HER-2/neu-specific delivery of HSP70 or its fragment to the surface of tumor cells using a two-module construct, with the barnase:barstar pair employed as a cohesive linker for protein modules. In this construct, the function of the first module carrying a high-specificity anticancer antibody and barnase consists in targeted binding to the surface of cancer cells. In its turn, barnase exposed on tumor cells due to this interaction acts as a site of selective binding between the second module consisting of barstar and HSP70 (or its fragment) and the target cells. In the approach being designed, the selective interaction between the first and the second modules is ensured by an unusually high constant of barstar binding to barnase. This protein heterodimer forms a complex with *K*_d_ ~ 10^-14^ M, which is comparable only to that of the streptavidin– biotin system (*K*_d_ ~ 10^-15^ M). In our previous studies, we have proved that the barnase:barstar complex shows a high potential as an agent for the targeted delivery of various drugs to tumor cells [36-39].

## EXPERIMENTAL

**The principles of building two-module molecular constructs for the targeted delivery of HSP70 to tumor cells**

**Fig. 1:**
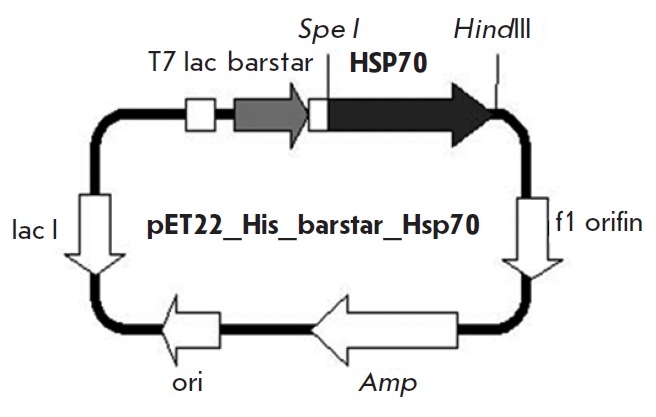
Scheme of the plasmid pET22_His_barstar_Hsp70 encoding the second module of the molecular construct carrying the Hsp70 effector protein. T7lac is the early promoter of bacteriophage T7 RNA polymerase; *HSP70 *is the gene encoding Hsp70; *Amp *is the ampicillin resistance gene

In order to build a supramolecular complex containing the HSP70 protein and the targeting mini-antibody, the barnase:barstar module had to be used to bind HSP70 to one of its components, barstar. It is known from experimental data [10] that the C-terminal domain of HSP70 is responsible for the stimulation of the cytotoxic and proliferative activities of NK cells. Therefore, the C-terminus of HSP70 in the recombinant protein being constructed had to remain unbound and accessible for interaction with natural killer cells, while barstar had to be attached to the N-terminus via a flexible peptide linker ensuring unrestricted rotation of functional domains in the target recombinant protein. These theoretical considerations were taken into account when constructing a plasmid encoding the target recombinant protein His6-barstar-HSP70, which consisted of the HSP70 protein (the inducible form of human HSP70 – Hsp70) linked to barstar with its N-terminus via the hinge peptide of human immunoglobulin IgG3 (ThrProLeuGlyAspThrThrHisThrSerGly) and carrying the hexahistidine tail at its N-terminus (*Fig. 1*). Similar procedures were conducted to build the second variant of the effector module that carried the 16 kDa C-terminal Hsp70 fragment (His6-barstar- Hsp70/16), instead of the full-length Hsp70 molecule. The previously designed 4D5 scFv-dibarnase construct [36] was used in this study as the first (targeting) module carrying specific anti-HER2/neu mini-antibodies.

**Cultures of tumor target cells**

SKOV3 human ovarian adenocarcinoma cells and BT- 474 human breast carcinoma cells overexpressing the HER-2/neu antigen were chosen as the target cells to be treated with the designed constructs. The cells were cultured in 6-well plates (Nunk, USA) and in 25 cm^2^ cell culture flasks (Costar, USA) in a RPMI 1640 medium (Flow Laboratories, UK) supplemented with 10% fetal calf serum (FCS), 50 μg/ml of streptomycin (Sintez, Russia), and 50 μg/ml of penicillin (Biosintez, Russia) in 5% CO_2_ at 37°C. The adherent cells attached to the culture flask substrate were removed from the substrate using a Versene solution. Human embryonic kidney cells (HEK 293) cultured under identical conditions were used as the control.

The expression level of the tumor-associated HER- 2/neu antigen on the surface of the cultured cell lines was tested using fluorescence microscopy and the previously designed recombinant constructs for visualizing cancer cells expressing the HER-2/neu antigen (anti-HER2/neu mini-antibody-barstar●GFP-barnase) [36, 37]. It has been demonstrated that the cell culture samples being used are characterized by a sufficiently high level of expression of the HER-2/neu surface antigen (the data are not shown).

**Treatment of the target cells with the designed constructs**

Hsp70 and its fragment, Hsp70/16, were delivered to tumor cells as the components of barstar-Hsp70 and barstar-Hsp70/16 recombinant proteins. At the first stage of targeted delivery, anti-HER2/neu mini-antibody (4D5 scFv protein) within the first module of the designed supramolecular complex was bound to the respective tumor-specific antigen on the cell surface (20 μg/ml, 60 min). Next, the barstar-Hsp70 and barstar-Hsp70/16 recombinant proteins were also strongly adsorbed onto the cell membrane (50 μg/ml, 60 min) due to the barnase:barstar interaction.

**Assessment of the efficiency of the binding between the designed constructs and target cells**

Flow cytofluorimetry was used to assess the efficiency of the targeted delivery of the heat shock protein to the surface of the target cells. The samples of the cells that had interacted with the first and second modules of the designed supramolecular complex were stained according to the conventional procedure [14] using BRM22 antibodies (Sigma, USA) specific to the C-terminus of HSP70 and anti-mouse IgG-FITC (Sigma, USA) as the second antibodies. The measurements were performed on a FACScan laser flow cytometer (Becton Dickinson, USA). At least 10,000 cells were analyzed for each sample. The statistical analysis was performed using the WinMDI software for processing the histograms recorded during the cytofluorimetric analysis.

Laser scanning confocal microscopy was used to visualize the targeted delivery of Hsp70 and its Hsp70/16 fragment to the surface of the tumor target cells. In these experiments, the target cells sequentially treated with the first and second modules of the designed constructs were stained with anti-HSP70 antibodies and second antibodies conjugated to AF488 fluorochrome (Molecular Probes, USA) using the conventional staining procedure. The cell precipitate obtained after centrifugation was placed onto a microscope slide; a specialized Mowioll gel-like polymerizable medium (Biomeda, USA) retaining cell morphology and preventing fluorochrome photobleaching was subsequently applied. The microscope slide was covered with a coverslip and left in the dark until the microscopic analysis. The photographs of the cells were taken on an ECLIPSE TE2000-E confocal microscope (Nikon, Japan).

**Assessment of the effect of treating tumor cells with the designed constructs on the cytotoxic activity of NK cells against these target cells**

NK cells isolated from human peripheral blood were used as cytotoxic effector cells in a series of *in vitro *experiments conducted to analyze the antitumor effect of the designed constructs. The magnetic separation technique using an NK cell isolation kit (MACS NK cell isolation kit II, Miltenyi Biotec, Germany) was employed to isolate NK cells from the mononuclear cell fraction obtained by density gradient sedimentation of peripheral blood from donors. The level of NK cell-mediated cytotoxicity was evaluated by CytoTox96 non-radioactive cytotoxicity assay (Promega, USA) based on a quantification of the lactate dehydrogenase (LDH) released from the target cells due to the action of natural killers on tumor cells. The experiments were conducted in accordance with the manufacturer’s protocol. Each experimental point was recorded in three replicas. The ratio between NK cells and the target cells placed into the wells was 7:1. BT-474 cells added to the wells 4 h prior to the experiment and subsequently treated with the tested recombinant constructs were used as the targets. At each stage of this procedure, after the addition of the components of the supramolecular complex in the wells and subsequent incubation of the target cells for 30 min at 4°C, the cells were precipitated by centrifugation. Supernatant was then removed, and the wells were washed to remove unbound recombinant proteins.

## RESULTS AND DISCUSSION

**Fig. 2:**
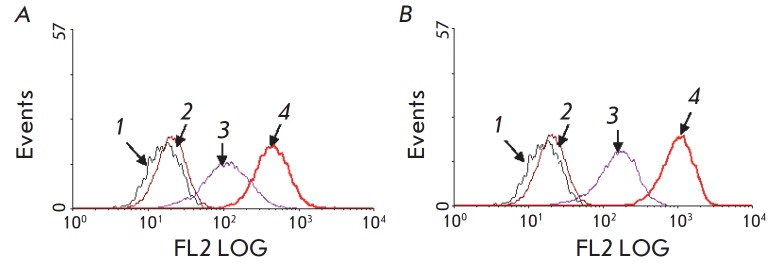
Cytofluorimetric analysis of the binding of the 4D5 scFv-dibarnase:barstar-Hsp70 (*A*) and 4D5 scFv-dibarnase:barstar-Hsp70/16 (*B*) complexes to the surface of BT-474 tumor cells. The cells were incubated with the first and second modules, and the samples were stained with the first anti-HSP70 antibodies (BRM22) and second FITC-labeled antibodies. X axis – fluorescence intensity; Y axis – number of events. The histograms: *1 *– autofluorescence control; *2 *– 4D5 scFv-dibarnase control; *3 *– barstar-Hsp70 (*A*) or barstar-Hsp70/16 (*B*) control; *4 *– 4D5 scFv-dibarnase:barstar-Hsp70 (*A*) or 4D5 scFv-dibarnase:barstar-Hsp70/16 (*B*).

The cytofluorimetric analysis demonstrated that the designed constructs can efficiently deliver Hsp70 and Hsp70/16 to the surface of tumor target cells. Similar findings characterizing the binding of Hsp70 and Hsp70/16 to the cell surface were obtained in experiments with the BT-474 and SKOV3 cell lines. Hence, below we summarize the results of the interaction between the designed constructs and BT-474 cells. The components of the 4D5 scFv-dibarnase:barstar- Hsp70(Hsp70/16) supramolecular complex efficiently binded to the cell surface: 4D5 scFv-dibarnase binded to the p185HER2 antigen, followed by interaction of barstar-Hsp70(Hsp70/16) with 4D5 scFv-dibarnase (*Fig. 2*). Furthermore, our findings indicate that the barstar- Hsp70 and barstar-Hsp70/16 proteins can independently interact with the cell membrane, leading to a shift in the histogram peaks of the respective control samples towards higher fluorescence signals. According to the literature data, some types of tumor cells can adsorb exogenous HSP70 onto their surface [15, 16].

**Fig. 3:**
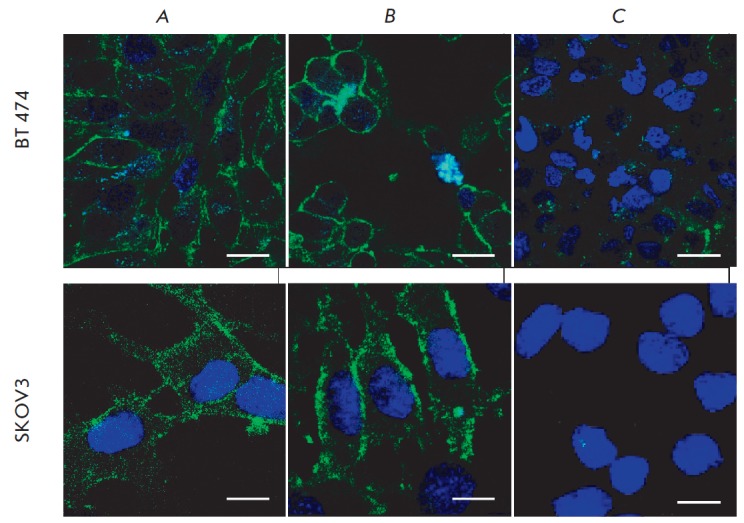
Visualization of the interaction between the developed constructs and two types of tumor cells. Upper row: BT-474 cells were treated with 4D5 scFv-dibarnase:barstar-Hsp70 (*A*) or 4D5 scFv-dibarnase:barstar-Hsp70/16 (*B*). Bottom row: SKOV3 cells were treated with 4D5 scFv-dibarnase:barstar-Hsp70 (*A*) or 4D5 scFv-dibarnase:barstar-Hsp70/16 (*B*). The treated cell samples were stained with the first anti-Hsp70 antibodies (BRM22) and second Alexa Fluor 488-labeled antibodies. *C *– control cell samples stained only with the second antibodies. Cell nuclei were stained with DAPY. The images were recorded using an ECLIPSE TE2000-E laser confocal microscope (Nikon, Japan). Scale bars, 10 µm

BT-474 cells expressed the p185HER2 antigen targeted by the mini-antibody in the first module. HEK 293 human embryonic kidney cells were used as a control for nonspecific binding of 4D5 scFv-dibarnase to the cell surface. The cytofluorimetric analysis showed that nonspecific binding to the cell membrane was observed for neither the first (4D5 scFv-dibarnase) nor the second (barstar-Hsp70(Hsp70/16)) module (the data are not shown).

Hence, the results indicate that the barnase:barstar systems ensure highly specific and efficient delivery of constructs carrying Hsp70 or its C-terminal fragment to the surface of tumor cells expressing the HER2/neu marker.

The efficiency of using the designed constructs for a targeted delivery of Hsp70 and its fragment, Hsp70/16, to the surface of BT-474 and SKOV3 cells was visualized by laser confocal microscopy. The target cells sequentially treated with the first and second modules of the designed constructs were stained with anti-HSP70 antibodies and the second antibodies conjugated to AF488 fluorochrome using the conventional staining procedure. The level of fluorescent staining was analyzed on an ECLIPSE TE2000-E confocal microscope. The results confirmed that Hsp70 and Hsp70/16 were present on the surface of the treated target cells (*Fig. 3*).

**Fig. 4:**
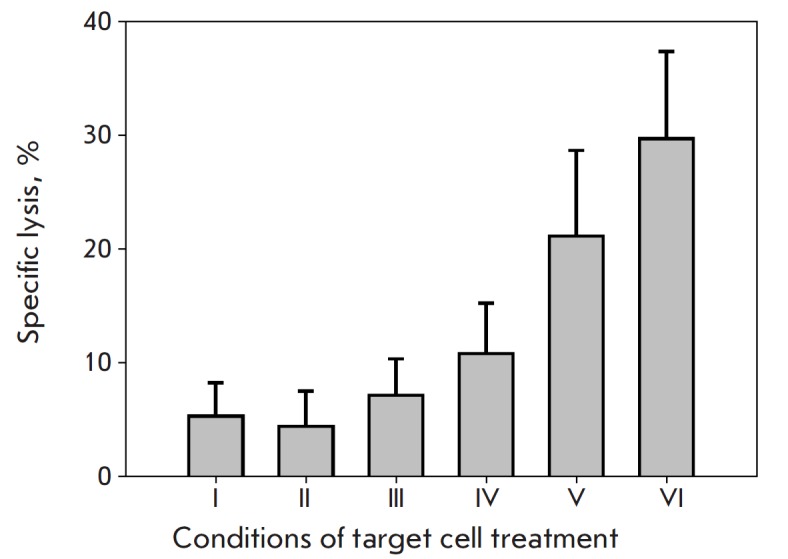
*In vitro *analysis of the effect of treating tumor target cells (BT-474) with the developed agents on the cytolytic activity of NK cells. I – control (no treatment); II – target cells treated with 4D5 scFv-dibarnase; III – target cells treated with barstar-Hsp70/16; IV – target cells treated with barstar-Hsp70; V – target cells treated with 4D5 scFv-dibarnase:barstar-Hsp70/16; and VI – target cells treated with 4D5 scFv-dibarnase:barstar-Hsp70

The influence of the designed constructs on the activation of cytotoxic effectors of the immune system was studied in the *in vitro *model of interaction between NK cells and the target tumor cells. BT-474 cells were used as the targets. Assessment of the interaction between effector cells and the target cells in this model demonstrated that targeted delivery of both the Hsp70 and Hsp70/16 molecules to the surface of tumor cells significantly enhances the antitumor cytolytic effect of NK cells. In our experiments, targeted delivery of the full-length Hsp70 molecule and its C-terminal fragment, Hsp70/16, to BT-474 cells enhanced the cytolytic effect of NK cells by more than five- and fourfold, respectively. Treatment of the target cells with individual components of the designed supramolecular complex (4D5 scFv-barnase acting as the “targeting” module and barstar-Hsp70 and barstar-Hsp70/16 acting as “effector” modules) did not significantly influence the cytolytic effect of NK cells. These findings are shown in *Fig. 4*.

## CONCLUSIONS

We have demonstrated that the designed two-module molecular construct was efficient in a targeted delivery of molecules that activate the cytotoxic effectors of the immune system (heat shock protein Hsp70 and its C-terminal fragment) to tumor target cells. The approach proposed in this study can underlie the design of novel agents for antitumor immunotherapy.

## Abbreviations

HSP70: 70 kDa heat shock protein
Hsp70: inducible form of human HSP70
Hsp70/16: 16 kDa C-terminal fragment of Hsp70
4D5 scFv: anti-HER2/neu mini-antibody
